# Parturition without Pain

**Published:** 1872-06

**Authors:** 


*Parturition without Pain, a Code of Directions for escaping the Primal Curse.* Edited by M. L. Holbrook, M. D., Second Edition. New York: Wood & Holbrook 1871.

Parturition without pain would indeed be a boon of inestimable value to mankind. The rules promulgated by the author for following a more Hygienic mode of living and dressing, if followed by the women of our land would, without doubt, produce more health and comfort if not less pain in child birth. The fashionable follies of the over heated drawing and ball room, of late suppers and indigestible food, and the foolish and hurtful mode of dressing are slowly but surely producing their certain effects of weakness and disease upon the women of our day.

The rules given by Dr. Holbrook are plain and simple, and the results cited are truly encouraging. Yet without doubt no rules of this or of any other character are capable of abolishing pain from the lying in chamber.

